# Supplementary material for: Headwaters to valley: Water quality in rivers transitioning from forest to agricultural bottomland
Source: PLoS One. 2025 Oct 30;20(10):e0316514. doi: 10.1371/journal.pone.0316514 (PMC12574886; doi:10.1371/journal.pone.0316514)
Supplement: S1 Table — (DOCX) [file pone.0316514.s001.docx]

**S1 Table. Dates and flow categories of transect sampling events.**

| **River** | **Date** | **Flow category** |
| --- | --- | --- |
| Dry River | 2/9/2020 | high |
| Briery Branch | 3/8/2020 | low |
| North River | 10/24/2020 | low |
| Briery Branch | 11/8/2020 | medium |
| Dry River | 11/21/2020 | medium |
| Briery Branch | 12/31/2020 | high |
| Dry River | 1/23/2021 | medium |
| Briery Branch | 3/13/2021 | high |
| North River | 3/20/2022 | medium |
| Dry River | 4/3/2022 | medium |
| North River | 6/7/2022 | medium |
| Dry River | 7/20/2022 | low |
| Briery Branch | 7/21/2022 | low |
| North River | 8/3/2022 | low |
| Dry River | 8/19/2022 | low |
| North River | 9/4/2022 | low |
| Briery Branch | 6/30/2023 | medium |
| North River | 7/3/2023 | medium |
| Dry River | 7/5/2023 | medium |
| North River | 5/22/2024 | high |
| Dry River | 5/23/2024 | high |
| Briery Branch | 5/23/2024 | high |
| North River | 6/24/2024 | low |
| Dry River | 6/24/2024 | low |
| Briery Branch | 6/24/2024 | low |
