# Supplementary figures and images for: Headwaters to valley: Water quality in rivers transitioning from forest to agricultural bottomland
Source: PLoS One. 2025 Oct 30;20(10):e0316514. doi: 10.1371/journal.pone.0316514 (PMC12574886; doi:10.1371/journal.pone.0316514)

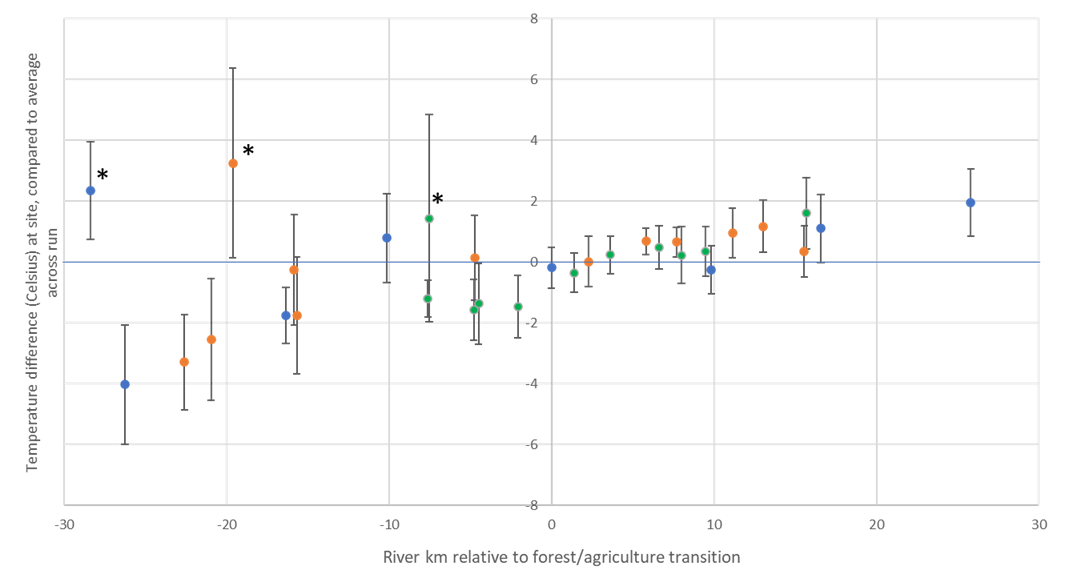

Supplement: S1 Fig — Temperature differential for each sample calculated as site temperature minus average temperature across its run (samples for the one river and one date). Blue, orange and green points represent parameter mean ± standard deviation values for the North River, Dry River, and Briary Branch, respectively. Asterisks with higher temperature differentials are pond or reservoirs. (TIF) [file pone.0316514.s002.tif]
